# Paradoxical pharmacological dissociations result from drugs that enhance delta oscillations but preserve consciousness

**DOI:** 10.1038/s42003-023-04988-8

**Published:** 2023-06-20

**Authors:** Joel Frohlich, Pedro A. M. Mediano, Francesco Bavato, Alireza Gharabaghi

**Affiliations:** 1 Institute for Neuromodulation and Neurotechnology, University Hospital and University of Tuebingen, Tuebingen, Germany; 2 Department of Computing, Imperial College London, London, UK; 3 Department of Psychology, University of Cambridge, Cambridge, UK; 4 Experimental and Clinical Pharmacopsychology, Department of Psychiatry, Psychotherapy, and Psychosomatics, Psychiatric University Hospital Zurich, University of Zurich, Zurich, Switzerland

**Keywords:** Neuroscience, Neurology

## Abstract

Low-frequency (<4 Hz) neural activity, particularly in the delta band, is generally indicative of loss of consciousness and cortical down states, particularly when it is diffuse and high amplitude. Remarkably, however, drug challenge studies of several diverse classes of pharmacological agents—including drugs which treat epilepsy, activate GABA_B_ receptors, block acetylcholine receptors, or produce psychedelic effects—demonstrate neural activity resembling cortical down states even as the participants remain conscious. Of those substances that are safe to use in healthy volunteers, some may be highly valuable research tools for investigating which neural activity patterns are sufficient for consciousness or its absence.

## Abstract (web summary)

A mini-review reflects on the use of pharmacological drug challenges to explore EEG delta oscillations which persist despite the preservation of human consciousness.

## Introduction

Neural oscillations in the delta (1–4 Hz) and slow (<1 Hz) frequency bands are generally indicators of unconsciousness^1–3^ or severely reduced consciousness^4^, especially when activity is diffuse and of high amplitude. Nonetheless, some pharmacological agents are known to enhance activity at these frequencies while sparing consciousness. Such paradoxical pharmacological dissociations (PPDs) are useful for falsifying putative spectral electroencephalogram (EEG) markers of conscious and unconscious states, e.g., if a substance can induce cortical slow waves during wakeful consciousness, then slow waves are not a universal indicator of loss of consciousness. Furthermore, understanding how such PPDs are possible is crucial for understanding how neural activity relates to consciousness.

A recent review^5^ described a range of conditions in which high amplitude delta oscillations (HADOs) appear in the awake and conscious state including, most notably, Angelman syndrome, a rare genetic condition characterized by diffuse HADOs during conscious wakefulness in children^6^. However, the above review focused largely on neurological conditions and less so on pharmacological manipulations. Here, we continue reviewing paradoxical EEG patterns by describing pharmacological drug challenges that induce HADOs without loss of consciousness. Our mini-review comprehensively covers literature in both non-human animals and human participants from over 70 years ago to the present day. By doing so, we illuminate PPDs in which the oscillatory regime exhibited by the EEG may be safely and reversibly switched independently of consciousness in the laboratory, e.g., as a means of validating candidate biomarkers of consciousness. Given their mechanistic similarities and the lack of meaningful boundaries between frequency bands^7^, we treat both delta and slow oscillations as belonging to the delta band for the purpose of this mini-review; thus, our principal focus is on oscillations occurring at frequencies at or below 4 Hz. Due to the inverse relationship between oscillatory frequency and spatial extent^7^, delta activity is often extensive across the scalp. For this reason, our mini-review does not particularly emphasize the scalp location of delta (but see the column “Spatial topography” in Table 1).

**Table 1:** Key EEG and MEG studies of pharmacologically-induced delta activity in humans.

Substance	Study	Sample size	Dose	Findings	Spatial topography
Carbamazepine (Antiepileptic)	Besser et al.^17^	*N* = 16	400 mg	Increase in delta (1.0–3.75 Hz) and theta (4.0–7.75 Hz) power after 35 days	The EEG power spectrum was computed from channel O2. However, focal slowing was noted in four patients over anterior temporal or frontal regions.
	Matsuura et al.^18^	*N* = 12	Variable (retrospective study)	Diffuse delta activity was observed in schizophrenia. Although delta activity was also noted in 31 other schizophrenia patients not taking carbamazepine, the proportion of patients taking carbamazepine was greater in patients with diffuse delta activity (28%) than in those without (9%). The finding was based on visual inspection without a clearly defined frequency range.	“[The] topographic distribution [of delta activity] was usually diffuse but sometimes there was frontal predominance, and those [patients] with persistent asymmetry and amplitude pre-dominance were also excluded”
Tiagabine (Antiepileptic)	Barnett et al.^23^	*N* = 15	15 mg	Increase in 1–4 Hz delta MEG power with acute dose 3 h after drug administration.	Diffuse increase in delta power across cortex; some weak predominance over frontal areas can be qualitatively noted in Barnett et al.^23^.
	Darmani et al.^20^ (PCI results) Darmani et al.^109^ (Spatial topographies)	*N* = 12	15 mg	Broadband increase in EEG power (delta, theta, alpha, and beta) accompanied by PCI values intermediate between consciousness and unconsciousness. Note that carbamazepine was also tested (600 mg, n = 15) but did not significantly alter delta power or PCI values.	The delta (1–3 Hz) power increase appears diffuse regardless of the recording period or whether the tiagabine power change was referenced to baseline or placebo.
GHB (GABA_B_ergic)	Metcalf et al.^36^	*N* = 20	35–63 mg/kg	High amplitude delta waves when participants were briefly aroused from unconsciousness; the frequency range is not clearly reported.	A frontal topography was reported: “background pre-rolandic delta and theta was still present.”
	von Rotz et al.^37^	*N* = 20	20–35 mg/kg	Trend-level increase in 2–4 Hz delta power with low-dose GHB accompanied by decrease in 8–10 Hz omega complexity	Source-localization using eLORETA suggests that delta and theta oscillations emerge from the posterior cingulate cortex.
	Vienne et al.^33^	*N* = 13	30 mg/kg	GHB increased EEG power (4–5 Hz and other frequency bins up to 9 Hz) during waking from overnight sleep relative to placebo.	“The spectral results […] were based on the central EEG derivation C3-A3, the most common derivation analyzed in sleep studies. Analyses of frontal and occipital derivations (F3-A2 and O1-A2) yielded similar results (data not shown), indicating that the findings shown were not site specific.”
Baclofen (GABA_B_ergic)	Badr et al.^40^	*N* = 10	30 mg 3x per day for multiple days	Baclofen significantly increased 3.5-7.5 Hz power in male patients with mild depressive symptoms relative to pretreatment at the earliest recording (two days) and subsequent recordings (five days and 19 days). A trend-level increase of 1.5–3.5 Hz power was also observed after two days (channel T3-T5).	“Moderate to severe abnormality diffusely located with maximum in the central region on the right side and also in the temporal region bilaterally.”
	Vienne et al. ^33^	*N* = 13	0.35 mg/kg	Baclofen increased ~ 4–10 Hz EEG power during waking from a nap relative to placebo; thus, the effect appears to be better described as theta rather than delta enhancement.	Results were from channel C3-A3, same as GHB data from Vienne et al.^33^ (see above).
Scopolamine (Anticholinergic)	Sannita et al.^50^	*N* = 8	0.25 - 0.75 mg	Doses of 0.5 and 0.75 mg resulted in increased delta (0.5–3.5 Hz) and theta (4.0–7.5 Hz) relative EEG power.	A central-occipital delta power increase was reported.
	Kikuchi et al.^49^	*N* = 16	0.25 mg	Intramuscular scopolamine enhanced absolute power in the delta band (2.0–3.8 Hz), and also relative power in the theta band (4.0–5.8 Hz), 60 min post-injection.	The power increase occurred “mainly over the central and parieto-occipital regions”.
DMT (Psychedelic tryptamine)	Timmermann et al.^62^	*N* = 13	7–20 mg	Increased 1–4 Hz delta and 4–8 Hz theta power (eyes closed spontaneous EEG with aperiodic component subtracted) induced by injected DMT correlated with the intensity of the psychedelic experience.	The scalp topography appears roughly diffuse. The authors speculate that “theta/delta rhythmicity under DMT […] may have a deep (e.g. medial temporal lobe) source”.
	Timmermann et al.^15^	*N* = 17	20 mg	Widespread increases in 1–4 Hz delta power during DMT in the context of concurrent fMRI while wearing an eye mask. Dynamic analyses also revealed positive correlations between delta power and minute-by-minute intensity ratings, as well as EEG signal complexity (Lempel-Ziv).	Frontal delta power correlated with increases in global fMRI connectivity, including “frontal, temporal, parietal, motor, and visual association cortices” in a subsample of 12 participants with usable fMRI and EEG data.
	Pallavicini et al.^64^	*N* = 35	~35 mg (estimated)	Increased delta (1–4 Hz) power (eyes closed spontaneous EEG) when DMT is inhaled in a naturalistic setting, accompanied by increases in gamma and decreases in alpha power.	Power increases in the delta band were “restricted to electrodes located primarily in occipital, parietal, and anterior-central regions.”

This table excludes non-human animal studies, as consciousness is more easily assessed in humans.

*GHB* gamma-hydroxybutyrate, *DMT* *N*,*N*-dimethyltryptamine, *PCI* perturbation complexity index.

Depending on the location of the reference electrode, either the peak or trough of each oscillatory cycle in the delta band corresponds to a down state of cortical silence^8^. During these periodic down states, cortical neurons switch to a more hyperpolarized resting membrane potential, increasing the difficulty of spiking and communicating with other neurons^9,10^. Conversely, up states result in a widespread spiking of cortical neurons at the opposite phase of the delta cycle. This dynamical regime can be represented by a simplified two-state model (described as the “monolithic brain” by Tononi^11^) in which the cortex is either up (spiking) or down (not-spiking).

Theories that attribute consciousness to the information-richness of cortical dynamics, such as integrated information theory in both its strong^12^ and weak^13^ formulations, explain the loss of consciousness that typically occurs under these conditions in terms of the loss of information integration. According to this view, HADOs may cause such loss of information by restricting the number of possible states visited by the brain. Yet even in rare genetic disorders where EEG oscillations are incongruent with the typical oscillatory regime seen during consciousness, informational measures (such as permutation entropy) reliably indicate the presence or absence of consciousness^14^. To learn how consciousness and complexity may persist under such unusual condition, one may use pharmacological manipulations that reversibly induce abnormal EEG patterns in healthy adults, thus allowing for comparisons of normal and abnormal cortical dynamics within the same individuals. The aim of such research should be to find a common denominator of consciousness^14^ across both normal and abnormal cortical dynamics.

Below, we review pharmacological manipulations that might be used for this purpose. Although the level consciousness is not entirely unimpaired by some substances (e.g., tiagabine) reviewed below, participants do not lose consciousness all together in the studies described, and in some cases, certain dimensions of consciousness are even enhanced (e.g., using psychedelic tryptamines). In the latter context, recent evidence^15^ shows that awake EEG delta activity correlates with global functional connectivity as assessed by concurrent functional magnetic resonance imaging (fMRI). Some substances which we review have potential as valuable experimental tools for revealing the common denominator of consciousness which persists even as an abnormal oscillatory regime emerges.

## Antiepileptic drugs

Antiepileptic drugs carbamazepine and tiagabine are reported to induce delta oscillations during wakeful consciousness, albeit while inducing mild sedation. Carbamazepine’s main mechanism of action is inhibition of voltage-gated sodium channels^16^. An early report of the delta-enhancing effects of carbamazepine came from a small study of patients who took a 400 mg dose over a period of 35 days, which reported increases in EEG delta and theta power relative to baseline^17^. Soon after, a similar effect was also inferred from an observational study of schizophrenia patients, many of whom regularly took carbamazepine with their antipsychotic medications and showed diffuse delta activity^18^. Although delta activity was diffuse, the authors noted that it was sometimes frontally predominant, similar to an earlier report^19^ of intermittent frontal rhythmic delta activity in eight schizophrenia patients taking antipsychotic drugs (exact medications not specified). However, a more recent study of acute carbamazepine (600 mg) in 15 healthy volunteers only found significant increases in resting EEG power in the theta, alpha, and beta bands^20^, and it is thus unclear if carbamazepine’s effect on EEG delta power is seen outside of chronic dosing.

**Fig. 1:**
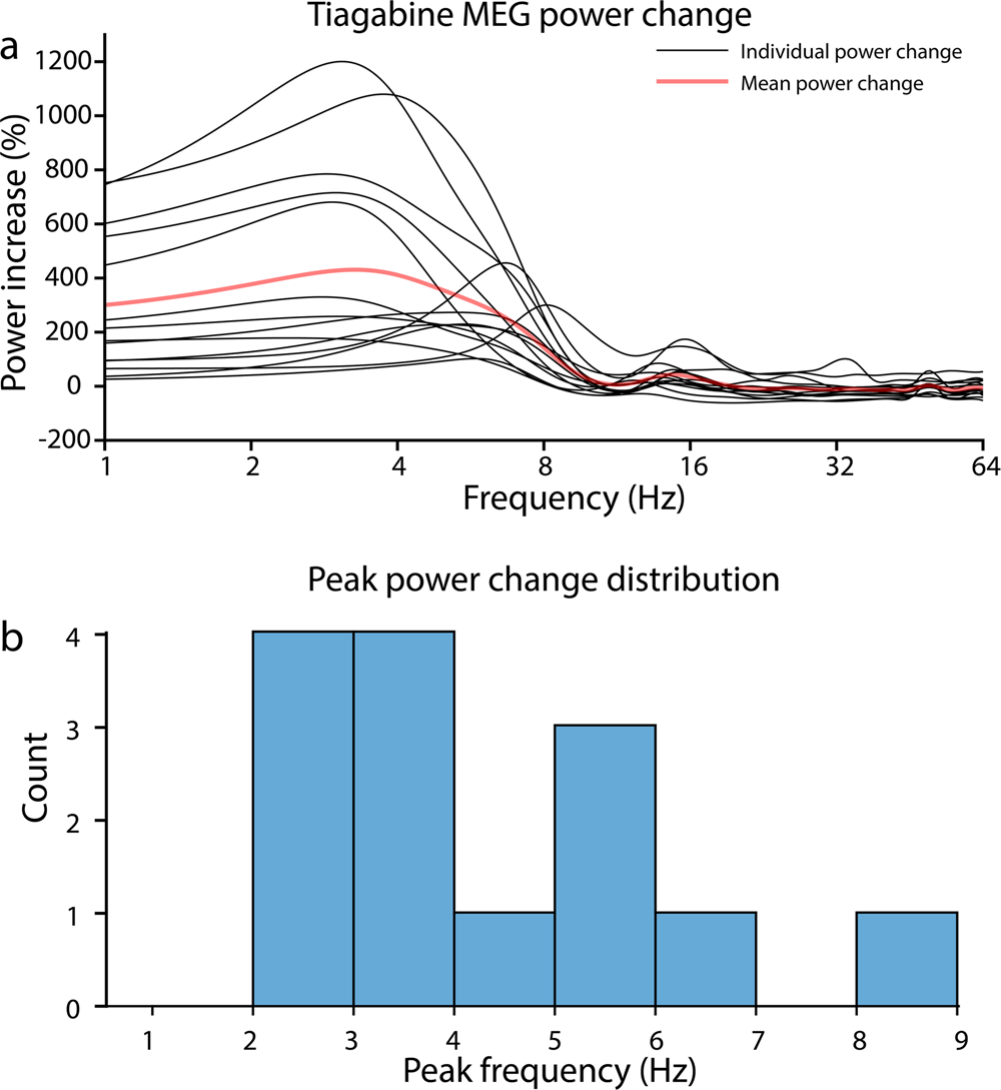
MEG power change (averaged across all sources and epochs) induced by tiagabine (15 mg) in 14 healthy volunteers. Data are from publicly available^26^ source-localized resting-state MEG recordings^23^. The experiment was approved by the UK National Research Ethics Service (South East Wales) and all participants gave written informed consent. One outlier participant was excluded due to an extremely large power increase for which we could not confirm that tiagabine was the sole cause. **a** Tiagabine induces clear increases in cortical oscillatory delta activity, as evidenced by the MEG power changes relative to placebo. The mean power change (red) induced by tiagabine is greatest at 3.3 Hz, featuring a more than 5-fold (430%) power increase from placebo. The largest power change we observed (after excluding one outlier, not shown here) was a 1200% power increase at 3.1 Hz. **b** The majority of participants (8/14) showed maximum power changes in the delta band (<4 Hz). The mean frequency of the maximum power change was 4.3 Hz, and the median frequency was 3.4 Hz. Source data supporting panel b are presented in Supplementary Data 1.

Unlike carbamazepine, tiagabine is a gamma-aminobutyric acid (GABA) reuptake inhibitor^21^. Besides enhancing delta power during wakefulness in young adult volunteers, as seen diffusely both in sensor space^22^ and source space^23^, tiagabine also induces episodes of hypersynchronous delta activity during wakefulness in rodents^24^, and in elderly humans, it increases delta power not only during NREM sleep, but also during REM sleep, i.e., when episodes of dreaming, and therefore some degree of consciousness, are common^25^. The degree of delta power enhancement induced by tiagabine during wakefulness in healthy volunteers, referenced to placebo, is highly variable, with some individuals exhibiting magnetoencephalogram (MEG) delta power increases >1000%^26^—comparable to the degree of delta enhancement in the Angelman syndrome EEG^27^—while others show much more modest changes (Fig. 1). Tiagabine is thus an attractive drug for studying PPDs that resemble the Angelman syndrome EEG phenotype, but its usefulness may be limited by tolerability issues^28^ (see below).

A recent small study by Darmani et al.^20^ investigated the “degraded state of consciousness” induced by tiagabine. Prior to each of four transcranial magnetic stimulation (TMS)-EEG sessions, 15 healthy volunteers were administered tiagabine, carbamazepine, brivaracetam (another antiepileptic drug), or placebo, experiencing each condition once. Similar to another report of partial sedation, confusion, disorientation, hallucinations, and amnesia caused by 15 mg tiagabine^28^, three participants had difficulty tolerating tiagabine and did not complete the subsequent TMS-EEG session. Tiagabine was found to significantly increase resting power in all frequency bands examined (delta, theta, alpha, and beta). Using the perturbational complexity index (PCI), a highly accurate measures of one’s level of consciousness based on the cortical response to TMS^29–31^, tiagabine was found to induce a PCI value (computed using the state transition algorithm^30^) intermediate between consciousness and unconsciousness. Similarly, other studies have shown that ketamine (not an antiepileptic, but rather an N-methyl-D-aspartate receptor antagonist) yields a PCI value that is intermediate between that typically seen in wakeful consciousness and unconsciousness, though it should be emphasized that PCI values obtained with ketamine were not significantly different from wakefulness in a small *n* = 6 sample^31,32^ (see the “other substances” section below for further discussion). By comparison, the other drugs investigated by Darmani et al.^20^ (brivaracetam and the ostensible delta-enhancer carbamazepine) did not significantly alter delta power or PCI values, though carbamazepine significantly increased power in other bands (theta, alpha, and beta). While both carbamazepine and tiagabine are generally known to increase delta EEG power (as shown in Fig. 1 for tiagabine), it is unclear whether results in the Darmani et al. study truly corresponded to delta-specific effects or rather non-specific increases in broadband power.

## GABA_B_ receptor agonists

Gamma hydroxybutyrate (GHB), sodium oxybate (i.e., the sodium salt of GHB), and baclofen are GABA_B_ receptor agonists that induce large increases in EEG delta power during sleep and wakefulness^33^. Unlike baclofen, GHB is also an agonist of the eponymous GHB receptor, an endogenous neurotransmitter^34^, and both a precursor and a metabolite of GABA^35^. One of the oldest published reports of pharmacologically induced HADOs is from a 1966 study of GHB’s effects in adult volunteers^36^. Volunteers given large doses (35–63 mg/kg) of GHB were awakened from an unconscious state induced by GHB and displayed HADOs while behaviorally responsive^36^. However, consciousness could only be maintained briefly (10–15 s) due to GHB’s strongly sedating effects. More recently, in an attempt to better understand the neural underpinnings of this PPD, GHB has been studied at lower doses that allow for uninterrupted wakefulness (20–35 mg/kg) in healthy male volunteers using sophisticated analyses such as source localization, global omega complexity, and functional connectivity analysis^37^. Findings of this analysis suggest that low-frequency oscillations induced by GHB emerge from the posterior cingulate cortex, a region that is sometimes implicated in consciousness due to its role in the default mode network, and a later study by the same group also reported that GHB increased delta power in posterior cingulate cortex, as well as medial prefrontal cortex, parahippocampal gyrus, and fusiform gyrus, during NREM sleep^38^. Results of both studies also suggested that GHB sedation may be related to increased lagged phase synchronization between posterior cingulate cortex and other regions^37,38^.

Likely because the above study of GHB chose a relatively low GHB dose, it only detected a trend-level increase in EEG delta power induced by GHB compared to placebo^37^. However, other studies from the 2010s^33,38^ have reinforced the results of older literature^36,39^, reproducing GHB-induced increases in relatively low-frequency EEG power during NREM sleep, REM sleep, and wakefulness. Evidence of enhanced activity at similar frequencies has also been obtained for baclofen—a similar GABA_B_ergic compound—in both humans^33,40^ and mice^41,42^, though most studies have focused on NREM sleep and suggest an effect in the theta band more so than the delta band. As with tiagabine^20^, GHB and baclofen are likely to be useful pharmacological tools for validating PCI and other biomarkers of consciousness under conditions of abnormal cortical dynamics, and both drugs may be better tolerated than tiagabine^28^, even when TMS is combined with pharmaco-EEG to compute PCI^43,44^. Underscoring the potential usefulness of these substances, GABAergic inhibition is a key parameter of perturbational complexity, as has been revealed by experiments that estimated PCI using electrical stimulation in ferret cortical slices while synchronous states (low PCI) were induced using both GABA_A_ and GABA_B_ receptor antagonists^45^. Interestingly, the above study induced low-frequency cortical oscillations by blocking GABA_B_ receptors, whereas baclofen and GHB induce low-frequency cortical oscillations while activating GABA_B_ receptors; thus, tuning the ratio of excitation to inhibition in either direction away from its optimum may induce hypersynchronous HADOs. However, because these experiments were performed in cortical slices, effects of GABA_B_ receptor antagonism on consciousness could not be studied.

## Anticholinergics

During states of wakefulness and arousal, acetylcholine acts as a neuromodulator and desynchronizes cortical activity^46^, thus diminishing delta oscillations. Conversely, anticholinergic drugs enhance the presence of delta oscillations. This has been noted for decades using the muscarinic antagonist atropine, which was first observed in 1952 to induce a PPD in dogs^47^. Several years later, a report also noted EEG slowing with 10 mg atropine in a small group of human volunteers, although the effect was reported to be more in the 5–8 Hz theta range^48^. Slowing effects have been noted using the muscarinic antagonist scopolamine in two small human studies that noted increased delta power^49–52^. This delta band effect was, however, not replicated by at least one EEG study^53^. More recently, a small study of healthy elderly participants found evidence of enhanced MEG connectivity (phase locking value) in the delta band under scopolamine^54^.

Because anticholinergic substances derived from the genus Datura, including atropine and scopolamine, are known to produce deliriant effects, cognitive impairment, and tachycardia, human research is highly limited. For instance, one study of scopolamine^50^ in healthy adult volunteers noted that following a 0.75 mg dose, participants “complained about subjective symptoms which were definitely unpleasant” and “were restless and confused, with mild muscular incoordination and evident drowsiness when not adequately aroused.” Due to these concerns, anticholinergic drugs—unlike the substances reviewed in previous sections—are not recommended for validating biomarkers of consciousness in humans. However, careful research using anticholinergic substances in non-human animals may reveal useful insights into the compatibility of delta oscillations with consciousness. A recent study of atropine and scopolamine in cats^55^, for example, found that the EEG pattern induced by anticholinergics, while resembling NREM sleep with respect to delta oscillations and sleep-like spindles, had a distinct profile in the gamma band, featuring gamma bursts and greater gamma coherence than NREM sleep. The authors concluded^55^ that these gamma EEG features, sometimes associated with consciousness^56,57^, might “explain why the animals remain awake in spite of the presence of slow waves and spindles.”

## Tryptamines

Of all substances that enhance delta oscillations while sparing consciousness, perhaps the most puzzling are the psychedelic tryptamines that actually increase the richness and intensity of visual imagery, imagination, and perceptual meaning^58,59^ while also inducing delta activity. This crucially demonstrates that, unlike some prior examples, delta-enhancing drugs do not necessarily induce drowsiness and partial-sedation. Most notable of these tryptamines is *N*,*N*-dimethyltryptamine (DMT), a principal ingredient in ayahuasca, a psychedelic brew made by the indigenous people of the Amazon basin^60^. DMT is also an endogenous neurotransmitter^61^, though its function as such is poorly understood. The first ever rigorous, placebo-controlled EEG study of DMT^62^ in humans recently revealed a surprising increase in delta power that occurred with intravenous DMT administration in 13 participants during eyes closed EEG. Moreover, this increase in delta power after subtracting the 1/f background of the EEG power spectrum correlated positively with subjective ratings of both the visual and overall intensity of the DMT experience. EEG signal complexity measured using the Lempel-Ziv algorithm^63^ (a common proxy for the level of consciousness, e.g., used to compute PCI) increased despite the introduction of delta oscillations which might otherwise be expected to reduce signal complexity by increasing the regularity and predictability of the signal. The above EEG findings were recently replicated by the same research team in a newer study using 17 participants each given 20 mg of intravenous DMT during simultaneous fMRI while wearing an eye mask^15^. Notably, increases in frontal delta power following DMT infusion correlated with increases in global fMRI connectivity. This replication also demonstrated that EEG delta power in the experiment did not significantly correlate with visual analog scales of drowsiness (in fact, the sign of the correlation coefficient was always negative). Separately, a study of inhaled DMT in naturalistic settings outside of the laboratory using 35 participants^64^ also reported an increase in delta power induced by DMT compared with an eyes closed resting baseline condition (as in the work by Timmerman et al.^62^, participants kept their eyes closed through the experiment). Interestingly, DMT enhanced gamma power and coherence relative to the baseline recording, thus suggesting a similar mechanism as that hypothesized for scopolamine^55^, whereby gamma-band activity sustains consciousness despite background delta activity. Finally, DMT in a naturalistic setting^64^ also recapitulates the laboratory finding of increased Lempel-Ziv complexity with DMT compared to the eye closed baseline EEG.

Other tryptamines have also been reported to increase delta power despite their psychedelic effects. In mice^65^, the DMT derivative 5-methoxy-DMT enhances theta and gamma power in medial prefrontal cortex and delta power in primary visual cortex. The latter finding supports the hypothesis^5^ that enhanced delta power observed in powerful psychedelic states relates to hyperpolarization of primary visual cortex when decoupled from the external sensory environment^66^, as may also occur in non-pharmacological dream states during REM sleep^67–69^ (though the recent findings of Timmermann et al.^15^ may point instead toward global hyperconnectivity). Additionally, 4-hydroxy-DMT (better known as psilocin, the pharmacologically active agent of psychedelic mushrooms) enhances 4 Hz power in mice during both wakefulness and REM sleep^70^; the latter effect was interpreted by the study’s authors as “a bleeding of NREM-like activities (delta waves, spindles, reduced gamma) into REM sleep.” Despite this, psilocybin (the prodrug of psilocin) does not appear to enhance delta MEG power^23^ or alter PCI values^71^ in human volunteers.

## Other substances

Besides the substances reviewed above, we also wish to highlight possible effects of other miscellaneous substances on delta EEG activity, albeit with caveats.

Subanesthetic doses of ketamine, a dissociative drug^72^, are known to induce a 1–3 Hz rhythm in layer 5 of retrosplenial cortex in mice (also demonstrated with the dissociative compound phencyclidine; here, the term “dissociative” refers to detachment or dissociation from one’s body or surroundings, rather than a PPD)^73^. Similarly, a 3–4 Hz rhythm in posterior cingulate and isthmus cingulate cortex was recently demonstrated using subanesthetic ketamine in human epilepsy patients^74^. Note that, as with ketamine, posterior cingulate cortex has also been identified as a plausible source of delta activity induced by GHB^37,38^. Delta frequency rhythms induced by ketamine, while linked to dissociation in both mice^73^ and humans^74^, appear limited to deep posteromedial areas observable only with invasive recordings—no enhancement of delta activity is seen in humans with subanesthetic doses of ketamine using noninvasive recordings, e.g., EEG^75,76^ or MEG^23^. In fact, at least one study reported a significant delta power decrease caused by subanesthetic ketamine in human EEG^77^. Studies which do report delta EEG activity in scalp recordings under ketamine have used anesthetic doses^78–81^.

Next, basmisanil, a selective GABA_A_-α_5_ negative allosteric modulator, enhances 4 Hz EEG power during wakefulness in patients with Down syndrome, while also suppressing power in the beta band^82^. However, in healthy participants, EEG power enhancement with basmisanil occurs more so in the theta band (6–9 Hz)^83^. By the same token, benzodiazepines (i.e., GABA_A_ positive allosteric modulators) generally have the opposite EEG effects—EEG suppression at delta/theta frequencies^83–86^ (but see an exception for delta here^77^) and enhancement at beta frequencies^77,83,84,87,88^—accompanied by partial or full sedation^89^. This demonstrates a very different PPD in which depressant and hypnotic effects occur without delta oscillations and are instead marked by high frequency activity more typical of cortical activation.

Another relevant substance is the acetylcholinesterase inhibitor donepezil. In healthy elderly adults, one study reported that an acute dose (5 mg) of donepezil caused a significant increases in EEG delta power alongside memory impairments^90^. On the other hand, chronic treatment with donepezil improves cognition and memory^91,92^ and reduces delta power during both wakefulness^93^ and REM sleep^94^ in patients with Alzheimer’s disease. While donepezil may have no obvious effect on waking consciousness, both it^95^ and another acetylcholinesterase inhibitor, galantamine^96^, affect sleeping consciousness by stimulating lucid dreaming, but this effect has not yet been studied in relation to EEG delta activity using acetylcholinesterase inhibitor compounds.

Near the end of our list are two sedating compounds, gabapentin and gaboxadol. Gabapentin, an analgesic and antiepileptic drug, is known to influence delta EEG activity. In rats, gabapentin normalizes deficits in delta power during slow-wave sleep induced by ethanol^97^. In humans, chronic daily use of gabapentin increases the percentage of delta power relative to a control group that received no drug or placebo^98^. However, it remains unknown whether gabapentin acutely enhances delta EEG power during wakefulness, especially at lower doses that do not require gradual titration. Next, gaboxadol is a muscimol derivative^99^ that has, like gabapentin, been shown to enhance low-frequency EEG power during NREM sleep^100^. In mice, gaboxadol administration leads to slow wave activity during wakefulness and REM sleep^101^. In humans, gaboxadol has been shown to enhance low-frequency power (including delta) during overnight wakefulness in the context of sleep restriction^102^. Delta enhancement has also been noted alongside enhancement of the theta and alpha bands in resting-state MEG recordings from male volunteers challenged with gaboxadol^22^. However, the amplitude of this gaboxadol-induced delta activity in humans is unclear and should be characterized by further studies.

Finally, opioids have been shown to increase delta EEG power^103,104^, but it is unclear to what extent this occurs in humans outside of anesthetic doses, and, moreover, this effect is inconsistent, with some studies reporting attenuation of delta activity by opioids^105,106^.

The effects of these and other substances on delta oscillations and consciousness warrant further investigation, as in many of the above cases, delta enhancement appears limited to a particular anatomical region, arousal state, or chronic dosing.

## Outlook and perspectives

Until recently, large delta and slow waves were commonly interpreted as evidence of unconsciousness. This interpretation is challenged by many documented exceptions, both from neurological disorders (reviewed previously^5^) and pharmacological manipulations. At first glance, some studies we have reviewed may appear to show that delta-enhancing drugs (e.g., tiagabine, GHB, scopolamine) simply induce drowsiness and partial-sedation. However, this seemingly uncomplicated relationship is contradicted by studies of psychedelic tryptamines such as DMT, which induce hypervivid experiences^59^ that often feel “more real than everyday normal consciousness”^107,108^. Future work is necessary to determine under what circumstances a gradual increase in delta activity may also indicate a gradual loss of consciousness and why this is not always the case (Box 1).

Although most pharmaco-EEG/MEG studies we cited used small samples (*N* ≤ 20, see Table 1), their findings are nonetheless sufficient to demonstrate that delta waves are in principle possible during consciousness. Because these substances allow PPDs to be observed in healthy volunteers, they may prove invaluable for future pharmaco-EEG experiments that seek to validate EEG biomarkers of conscious state (e.g., PCI) in neurologically-typical adults. In particular, GABA_B_ergic substances such as GHB may prove to be safe and well-tolerated models for abnormal cortical dynamics manifesting in the delta band. Following recent work that has used rare neurogenetic disorders to model abnormal cortical dynamics for the purpose of validating EEG biomarkers of consciousness^14^, such substances could be administered in healthy adult individuals to further validate EEG markers of consciousness such as the entropy of spontaneous EEG signals or perturbational complexity.

Box 1 Recent developments and outstanding questions **Recent developments**

• A variety of pharmacological agents enhance delta EEG activity while preserving or even elevating certain dimensions^62,64^ of phenomenological consciousness.

• A recent investigation of the psychedelic compound DMT using concurrent EEG-fMRI found correlations between frontal delta power and global fMRI connectivity, suggesting that global hyperconnectivity in the psychedelic state might explain accompanying delta oscillations.

• Subanesthetic doses of ketamine may yield enhancements of delta power in deep cortical regions^73,74^ that are not easily detected using noninvasive methods such as EEG^75^ or MEG^23^.

• The antiepileptic drug tiagabine induces increases in cortical delta MEG power up to 1000% or more relative to placebo in some individuals^26^ (Fig. 1), which is comparable to the extremely abnormal delta EEG phenotype seen in the genetic disorder Angelman syndrome^27^.

• The foregoing suggests that drugs like tiagabine with comparable levels of delta enhancement might be used to reversibly induce highly abnormal cortical dynamics in healthy participants in the laboratory, e.g., as a means of challenging candidate biomarkers of conscious state^14^.

• Toward this purpose, GABA_B_ergic drugs like GHB might be better tolerated than tiagabine^28^, though recent studies of the former^37^ have mainly explored relatively low doses that might be insufficient to achieve the above goal.

• A recent study^20^ successfully overcame tiagabine’s tolerability concerns and used it to challenge PCI as an approximation of conscious level, with a result showing intermediate PCI values between those that have been previously established for wakefulness and NREM sleep.

**Outstanding questions**

• Under what circumstances does a gradual increase in delta activity also indicate a gradual loss of consciousness?

• Why do some delta-enhancing drugs induce partial-sedation and drowsiness^36,50^ while others induce hypervivid psychedelic experiences^62^?

• To what extent do abnormal cortical dynamics induced by delta-enhancing drugs accurately simulate abnormal cortical dynamics in neurological or genetic disorders^14^ such as Angelman syndrome?

• Do PPDs between EEG oscillations and consciousness also extend to non-oscillatory EEG features (e.g., signal entropy) and consciousness?

• Do delta-enhancing drugs that spare consciousness always yield PCI values intermediate between consciousness and unconsciousness, as previously demonstrated with tiagabine^20^?

• Do awake delta oscillations correspond to global hyperconnectivity^15^ in other contexts besides DMT, e.g., PPDs induced by non-psychedelic drugs?

## Supplementary information

Description of Additional Supplementary Files

Supplementary Data 1

## Data Availability

Fig. 1 is based on previously published tiagabine MEG data which are publicly available through the Harvard Dataverse repository: https://dataverse.harvard.edu/dataset.xhtml?persistentId=doi:10.7910/DVN/9Q1SKM. The source data used to plot Fig. 1b are also provided in Supplementary Data 1.
