# Supplementary material for: Paradoxical pharmacological dissociations result from drugs that enhance delta oscillations but preserve consciousness
Source: Commun Biol. 2023 Jun 20;6:654. doi: 10.1038/s42003-023-04988-8 (PMC10282051; doi:10.1038/s42003-023-04988-8)
Supplement: Supplementary file 1 — Description of Additional Supplementary Files [file 42003_2023_4988_MOESM1_ESM.pdf]

Description of Additional Supplementary Files

File name: Supplementary Data 1

Description: Source data for Figure 1b
